# The origin of sound damping in amorphous solids: Defects and beyond

**DOI:** 10.1126/sciadv.adu6097

**Published:** 2025-04-11

**Authors:** Elijah Flenner, Grzegorz Szamel

**Affiliations:** Chemistry Department, Colorado State University, Fort Collins, CO 80523, USA.

## Abstract

Comprehending sound damping is integral to understanding the anomalous low temperature properties of glasses. After decades of studies, Rayleigh scaling of the sound attenuation coefficient with frequency, $\Gamma \propto \omega^{d+1}$, became generally accepted. Rayleigh scaling invokes a picture of scattering from defects. It is unclear how to define glass defects. Here, we use a particle level contribution to sound damping to determine areas in the glass that contribute more to sound damping than other areas, which allows us to define defects. Over a range of stability, sound damping scales linearly with the fraction of particles in the defects. However, sound is still attenuated in ultrastable glasses where no defects are identified. We show that sound damping in these glasses is due to nearly uniformly distributed non-affine forces that arise after macroscopic deformation. To fully understand sound attenuation in glasses, one has to consider contributions from defects and a defect-free background, which represents a different paradigm of sound damping in glasses.

## Teaser

Sound attenuation in glasses exhibits features associated with scattering from defects without the presence of defects.

## INTRODUCTION

The disordered structure of glasses results in low temperature properties that are different from those of crystals, and an explanation of this finding remains an extensive area of research (*1*, *2*). It was initially thought that low-frequency vibrations of glasses would be the plane waves of the Debye model, as they are in crystals. The seminal paper by Zeller and Pohl (*2*) showed that the low-temperature specific heat and thermal conductivity of glasses were inconsistent with the Debye model. Later, inelastic neutron scattering (*3*) and Raman scattering (*4*, *5*) showed that there is an excess in the density of states above the Debye prediction.

The excess vibrational density of states results in a peak in the reduced specific heat $C_{p}/T^{3}$ at a temperature between 3 and 15 K (*1*, *6*). In the same temperature range a plateau in the thermal conductivity appears (*1*, *2*), which suggests a connection between the two observations. To fit both the reduced specific heat peak and the thermal conductivity plateau with the same parameters, Yu and Freeman (*7*) found that they needed $\omega^{4}$ Rayleigh scaling of sound damping.

Rayleigh scaling invokes a picture of scattering from defects, but it is an ongoing question as to what constitutes a defect in a glass. Previous to Yu and Freeman, Zaitlin and Anderson (*8*) considered density fluctuations as defects but were unable to find reasonable values for Rayleigh scattering coefficients to quantitatively reproduce the thermal conductivity plateau. This observation leads to the question of the identity of the defects that are responsible for Rayleigh scaling.

A possible definition of a defect comes from the soft potential model (*1*). The soft potential model postulates the existence of excitations that couple to sound waves resulting in sound damping that scales as $\omega^{4}$ (*9*, *10*). Wang *et al.* (*11*) found that sound damping scales linearly with the density of quasi-localized vibrational modes, defined through the vibrational mode participation ratio (*12*, *13*), suggesting that quasi-localized modes may be scattering defects. However, they did not establish a direct connection between the quasi-localized modes and sound damping, and the question still remained whether the quasi-localized modes should be considered damping defects.

Some theories do not explicitly invoke defects and also predict Rayleigh scaling. Fluctuating elasticity theory describes glasses as elastic materials with spatially varying elastic constants. When the spatial extent of elasticity fluctuations is much smaller than the wavelength of the sound wave, fluctuating elasticity theory predicts Rayleigh scaling of sound damping. Since elasticity is a property of a bulk material, it is not immediately clear how to define it locally (*14*). For two-dimensional systems Kapteijns *et al.* (*15*) argued that sample-to-sample elasticity fluctuations can be used to determine the relative scaling of sound attenuation, but this still leaves the question of the identification of elastic defects associated with sound damping.

Mahajan and Ciamarra (*16*) examined this question. They found that local elastic constants defined in a specific way [see supplementary materials of (*16*)] have fluctuations that have the same dependence on a glass model’s potential cutoff as the sample-to-sample fluctuations examined in (*15*). They defined a coarse graining length that they identified with the characteristic defect size. They used this identification to show that correlated fluctuating elasticity theory (*17*–*19*), with one adjustable parameter, accurately captures the relative dependence of sound damping on glass properties.

Rayleigh scaling is also predicted by theories where there is no clear way to define defects. Euclidean random matrix model, which posits that an amorphous solid may be approximated as a set of randomly placed sites connected by harmonic springs, leads to Rayleigh scaling of sound damping (*20*–*22*). In this model, defects cannot be identified beyond individual sites.

Baggioli and Zaccone (*23*) developed an approximate theory that predicts Rayleigh scaling of sound damping without invoking the concept of defects. Their theory invokes an averaging procedure that smears out any explicit effect of defects.

All of the theories mentioned above provide a reasonable starting point and predict Rayleigh scaling of sound damping in three dimensions. However, they leave a confusing picture to the underlying damping mechanism and whether defects are necessary for Rayleigh scaling. To differentiate the different pictures, one needs a quantitatively accurate theory with no fitting parameters. Only then one can determine the role of defects in sound damping.

Here, we use a recently developed microscopic theory (*24*) that accurately predicts sound damping in the harmonic approximation with no adjustable parameters. Within many glasses, we are able to find areas that result in strong sound damping over the frequency range where we observe Rayleigh scaling. We identify these areas as sound damping defects. We find that the fraction of particles in these defects scales linearly with the Rayleigh scaling coefficient for a series of glasses with widely different stabilities. However, we find finite sound damping and Rayleigh scaling for exceptionally well annealed glasses where we do not find any defects, and thus, the defects cannot be the sole source of sound damping.

Sound damping without defects originates from small non-affine motions that occur due to displacements induced by the sound wave. These motions have to be present for any noncentro-symmetric structure. The resulting picture is that sound damping has a defect contribution on top of a defect-free background, which represents a different paradigm in the understanding of sound damping in glasses. Our theory quantitatively captures both effects.

## RESULTS

### Theory and simulation comparison

We study a two-dimensional glass forming polydisperse mixture of spheres interacting via a $r^{-12}$ repulsive potential in a fixed volume V. See Materials and Methods for details. Using the swap algorithm (*25*), our model system can be equilibrated down to temperatures below the mode coupling temperature of 0.123 and the estimated glass transition temperature of 0.082. Glasses of different stability are created by equilibrating a fluid and quenching it into an inherent structure. These glasses are labeled by the temperature from which they were quenched, which we refer to as the parent temperature $T_{p}$. The parent temperature is similar to the fictive temperature, and the lower the $T_{p}$ the more stable the glass.

To calculate sound damping in simulations and as a starting point of our theory, we consider a glass undergoing harmonic vibrations around an inherent structure. The equations of motion read

$$\partial_{t}^{2}u_{n}={\ddot{u}}_{n}=-\sum_{m}H_{\mathrm{nm}}\cdot u_{m}$$ (1)

where ℋ is the Hessian calculated at the inherent structure positions $\left\{R_{n}\right\}$, and $u_{n}$ is the displacement of particle n from $R_{n}$. More details on the sound damping theory and details of the calculation are found in Materials and Methods. The theory predicts that the attenuation coefficient of a transverse sound wave in the low-frequency limit can be expressed as

$$\begin{array}{c} \Gamma_{T}\left(\omega \right)=k^{2}\sum_{\omega_{p}}\delta \left(\omega -\omega_{p}\right)\frac{\pi }{2\omega_{p}^{2}}{\langle 1|X|\varepsilon \left(\omega_{p}\right)\rangle }^{2}=\\ {\left(\omega /v_{T}\right)}^{2}\sum_{\omega_{p}}\delta \left(\omega -\omega_{p}\right)\epsilon \left(\omega_{p}\right) \end{array}$$ (2)

In Eq. 2, 〈1∣X is the non-affine force field due to a deformation, $|\varepsilon \left(\omega_{p}\right)\rangle$ is an eigenvector of the Hessian ℋ corresponding to eigenfrequency $\omega_{p}$, and $v_{T}$ is the transverse speed of sound. Details of the calculation for finite systems can be found in Materials and Methods. We note that inner product ${\langle 1|X|\varepsilon \left(\omega_{p}\right)\rangle }^{2}$ features in the theory of Baggioli and Zaccone (*23*), which was developed independently at the same time as our theory. However, the complete Baggioli and Zaccone’s expression for sound damping differs from ours.

**Fig. 1.**
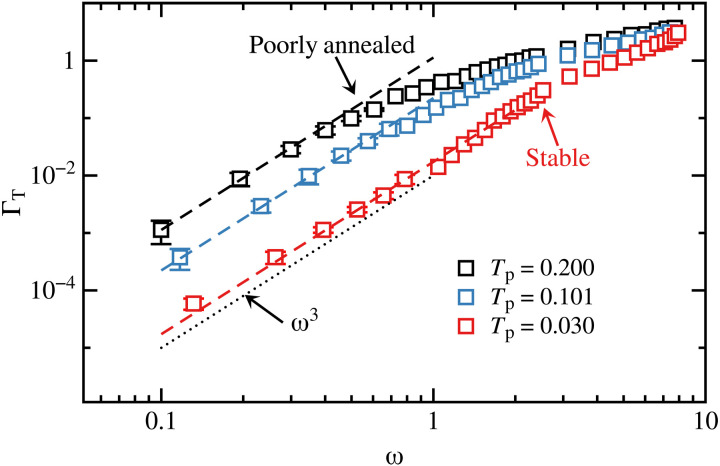
Theory and simulation comparison. Comparison of transverse sound damping $\Gamma_{T}$ calculated from simulations (squares) and the low wave vector expansion of the theory (dashed lines) as a function of frequency ω. Glasses at three different stability are shown. A poorly annealed glass, $T_{p}=0.2$ (black), a stable glass $T_{p}=0.101$ (blue), and an exceptionally stable glass $T_{p}=0.03$ (red). There is near perfect agreement between simulations and theory with no adjustable parameters. The dotted line represents Rayleigh scaling $\omega^{3}$, which is in good agreement with the results.

We find that sound damping calculated from simulations (squares) and theory (dashed lines) agree very well in the $\omega^{3}$ (dotted line) Rayleigh scaling region (Fig. 1). We emphasize that our theory has no adjustable parameters, and thus, it captures the relative change as well as the magnitude of sound damping. We compare the results of the calculation using Eq. 2 to fits of the function $\Gamma_{T}\left(\omega \right)=B_{3}\omega^{3}$ in Table 1.

**Table 1.** Damping coefficient determined using various methods. The first method, Fit, corresponds to $B_{3}$ obtained from fitting the squares shown in Fig. 1 to $\Gamma_{T}\left(\omega \right)=B_{3}\omega^{3}$. The second method uses the results obtained from Eq. 2. The third method uses ϵ(ω) averaged over the Rayleigh scaling regime.

*T*_p_	Fit, *B*_3_	Eq. 2	$A_{D}\bar{\epsilon }/c_{T}^{2}$
0.2	1.0 ± 0.2	1.2 ± 0.2	1.1 ± 0.3
0.101	0.19 ± 0.02	0.21 ± 0.02	0.22 ± 0.04
0.03	0.018 ± 0.003	0.015 ± 0.002	0.018 ± 0.004

**Fig. 2.**
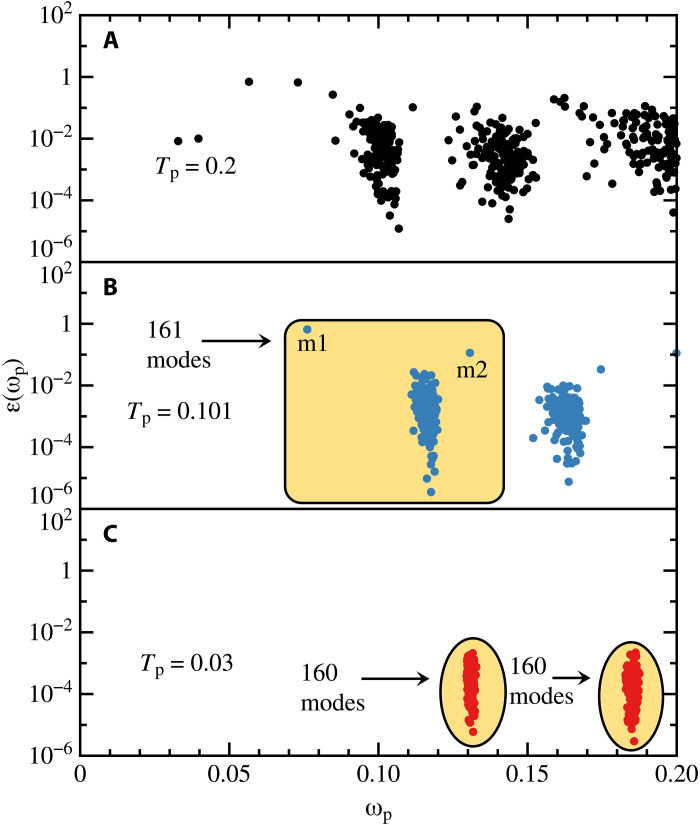
Vibrational mode level contribution to damping. The contribution to sound damping from different eigenvectors $|ℰ\left(\omega_{p}\right)>$ with a frequency $\omega_{p}$. (**A**) A poorly annealed glass, $T_{p}=0.2$. (**B**) A well annealed glass, $T_{p}=0.101$. (**C**) An exceptionally stable glass, $T_{p}=0.03$.

To examine the properties of the glass that give rise to sound damping, we calculate the mode level contribution to sound damping using $\epsilon \left(\omega_{p}\right)$. Shown in Fig. 2 are the values of $\epsilon \left(\omega_{p}\right)$ calculated at each parent temperature for 40 glass samples for the range of $\omega_{p}$ needed to determine sound damping for the smallest wavelength sound wave allowed due to periodic boundary conditions. We find a marked change of the mode level contribution with increasing stability, i.e., decreasing $T_{p}$.

For $T_{p}=0.2$ (Fig. 2A), there are clusters around frequencies corresponding to the first two transverse sound waves, but in addition, there are contributions at frequencies between these clusters. The value of $\epsilon \left(\omega_{p}\right)$ varies by several orders of magnitude for a given $T_{p}$. The overall scale of $\epsilon \left(\omega_{p}\right)$ decreases with decreasing $T_{p}$; it is much larger for $T_{p}=0.2$ than for $T_{p}=0.101$ and 0.03.

For $T_{p}=0.101$ (Fig. 2B), there are two distinct clusters of modes with three modes making a noticeably larger contribution than other modes. If the Debye model was an accurate description of the low-energy excitations, then each configuration should contribute four modes to the first transverse wave and four modes to the second transverse wave, which represents the two clusters in Fig. 2B. Since we used 40 configurations for the calculation, according to the Debye model, the first cluster should contain 160 modes. However, there are 161 modes in the highlighted box. Therefore, there is one discrete excess mode over the Debye model in this frequency range. We note that no other modes contribute to sound damping for the lowest-frequency sound wave than the modes shown in Fig. 2.

For $T_{p}=0.03$ (Fig. 2C), there are two distinct clusters with 160 modes as expected for the Debye model. For the 40 configurations we studied, there are no excess modes obtained from diagonalizing the Hessian in this frequency range. In addition, $\epsilon \left(\omega_{p}\right)$ is much smaller than for $T_{p}=0.2$. In the “Damping defects” section, we study what properties of the eigenvectors give rise to such large differences in $\epsilon \left(\omega_{p}\right)$.

### Damping defects

To examine whether there are areas of the glass that contribute to sound damping more than others, we examine a particle level contribution to damping. To this end, we write $\sqrt{N}\langle 1|X$ in terms of the contributions of individual particles, which we denote $\Xi_{n}^{\alpha }$

$$\sqrt{N}\langle 1|X=\left(\Xi_{1}^{x},\Xi_{1}^{y},\ldots ,\Xi_{n}^{x},\Xi_{n}^{y},\ldots ,\Xi_{N}^{x},\Xi_{N}^{y}\right)$$ (3)

The low-frequency sound damping is proportional to ${\left\{\sum_{n}\left[\Xi_{n}^{x}E_{n}^{x}\left(\omega_{p}\right)+\Xi_{n}^{y}E_{n}^{y}\left(\omega_{p}\right)\right]\right\}}^{2}$, where $E_{n}^{x,y}\left(\omega_{p}\right)$ are the components of the eigenvector corresponding to frequency $\omega_{p}$. We define the particle level contribution

$$C_{n}=\Xi_{n}^{x}E_{n}^{x}\left(\omega_{p}\right)+\Xi_{n}^{y}E_{n}^{y}\left(\omega_{p}\right)$$ (4)

The quantity $C_{n}$ can be positive or negative, and sound damping is given by the square of the sum of these contributions. Our hypothesis is that there exists spatial regions with large $|C_{n}|$, and these regions make a relatively large contribution to sound damping.

Two obvious reasons that could make $|C_{n}|$ large is that the magnitude of the vector $\Xi_{n}=\left(\Xi_{n}^{x},\Xi_{n}^{y}\right)$ is large or the magnitude of the vector $E_{n}\left(\omega_{p}\right)=\left[E_{n}^{x}\left(\omega_{p}\right),E_{n}^{y}\left(\omega_{p}\right)\right]$ is large. We will show that the eigenvectors for which there exist clusters of particles with large $|E_{n}\left(\omega_{p}\right)|$ have a large contribution to sound damping over a range of frequencies. We classify these particles as belonging to defects. We find that these defects can strongly influence sound damping, but they are not necessary for sound damping in glasses. Rayleigh scaling of sound damping can occur without defects.

It is instructive to examine the contributions to sound damping for our $T_{p}=0.101$ glass, (Fig. 2B), since there are two easy to identify modes that makes a large contribution to sound damping. We denote these mode as m1 and m2 in Fig. 2B. Both of these modes originate from the same configuration.

**Fig. 3.**
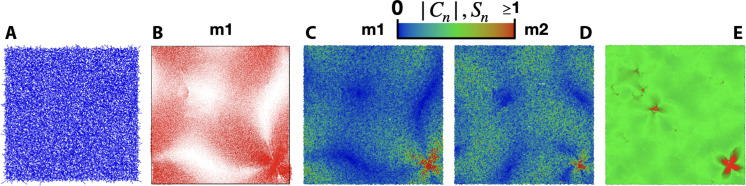
Visualization of damping defects. (**A**) A non-affine force field for a shear deformation of the configuration represented in (B) to (E). (**B**) The eigenvector, m1 in Fig. 2B, with the largest contribution to sound damping out of the 40 $T_{p}=0.101$ glasses we studied. An area of large $|E_{n}\left(\omega_{p}\right)|$ can be found in the lower right corner. (**C**) Color map showing the relative contribution to sound damping $|C_{n}|$ for the same vibrational modes shown in (B), which shows that the particles within area of large $|E_{n}\left(\omega_{p}\right)|$ make the largest contribution to sound damping for this vibrational mode. (**D**) The particle level contribution for the mode with the second largest contribution to sound damping for $T_{p}=0.101$ glasses (m2 in Fig. 2B), showing that the same soft spot influences sound damping for both modes at different frequencies. This mode originates from the same glass as m1. (**E**) A color map of $S_{n}$ using the same glass configuration as shown in (A) to (C). The defect particles identified using the procedure described in the text are colored red.

Shown in Fig. 3A is the non-affine force field due to a simple shear deformation for the configuration with the eigenvector with the largest contribution to sound damping (Fig. 3B), labeled m1 in Fig. 2B. There is no obvious regions of large $|\Xi_{n}|$. In contrast, there is a cluster of large $|E_{n}|$ in the eigenvector m1.

Shown in Fig. 3C is a color map of $|C_{n}|$ indicating the size of the contribution to sound damping for each particle corresponding to the mode shown in Fig. 3B. There is correspondence with the largest values of $|C_{n}|$ with the largest $|E_{n}\left(\omega_{p}\right)|$. In Fig. 3D, we show the particle level contributions to damping within the mode labeled m2 in Fig. 2B, which has the second largest contribution to the lowest-frequency sound wave and comes from the same configuration as m1. In the same region as for m1, the particle level contribution to sound damping is the largest.

One region of space can make a large contribution to sound damping over a range of frequencies. These regions can be considered defects with regard to sound wave propagation. Our theory predicts that these defects are large sources to sound damping. However, since the non-affine force field is nonzero everywhere in the glass, defect-free areas also contribute to sound damping.

To determine areas of the glass where there is strong damping, we want to find areas where $|E_{n}\left(\omega_{p}\right)|$ is larger than expected for a plane wave over a range of frequencies. If the eigenvector $|\varepsilon \left(\omega_{p}\right)\rangle$ is a plane wave, then ${|E_{n}\left(\omega_{p}\right)|}^{2}\leq 2/N$ for each particle. Rather than focusing on a single-frequency $\omega_{p}$, we consider the range of frequencies that encompasses the Rayleigh scaling regime for every $T_{p}$. For our system size, this results in the 24 lowest-frequency eigenvectors (excluding the uniform translations). Thus, we consider a particle part of a defect if $S_{n}=\left(N/2\right)\sum_{p=1}^{24}{|E_{n}\left(\omega_{p}\right)|}^{2}/24>1$. We define $w_{n}=1$ if a particle is within a defect and zero otherwise.

Shown in Fig. 3D is a color map of $S_{n}$ where the red regions correspond to defects in the configuration with the eigenvectors corresponding to m1 and m2 in Fig. 2B. The procedure clearly picks out the region of large damping seen in Fig. 3 (B to D) as well as some smaller regions.

**Fig. 4.**
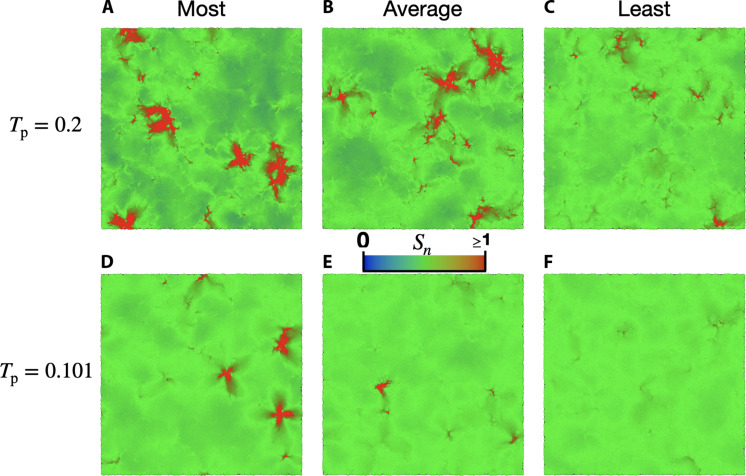
Stability dependence of defects. Stability and configuration dependence of defects. A color map of $S_{n}$ for the $T_{p}=0.2$ glass with (**A**) the most number of particles in a defect, (**B**) a glass with close to the average number of particles in a defect, and (**C**) the glass with the least number of particles in a defect. A color map of $S_{n}$ for the $T_{p}=0.101$ glass with (**D**) the most number of particles in a defect, (**E**) a glass with close to the average number of particles in a defect, and (**F**) a glass with the least number of particles in a defect. Three glasses had zero defects for $T_{p}=0.101$. There were no defects for our $T_{p}=0.03$ glasses.

For each parent temperature, we can determine the glass configuration with the largest number of particles within defects and the least number of particles within defects. For our $T_{p}=0.2$ glasses, we identified a defect within every glass configuration. Shown in Fig. 4A is a color map of $S_{n}$ of the configuration with the largest number of particles within defects. Figure 4B shows a configuration with an average number of particles within defects, and Fig. 4C shows the configuration with the smallest number of particles within defects for the $T_{p}=0.2$ glasses.

For our $T_{p}=0.101$ glasses, defects can be found in 37 of the 40 glass configurations. Shown in Fig. 4D is a color map of $S_{n}$ for the configuration with the largest number of particles within defects, the configuration with about the average number of particles in defects is shown in Fig. 4E, and a configuration with the smallest number of particles in defect (zero) is shown in Fig. 4F. Visually, we can see a large change in the number and size of the defects with increasing stability. For our $T_{p}=0.03$ glasses, we did not find defects in any of the 40 glass configurations.

**Fig. 5.**
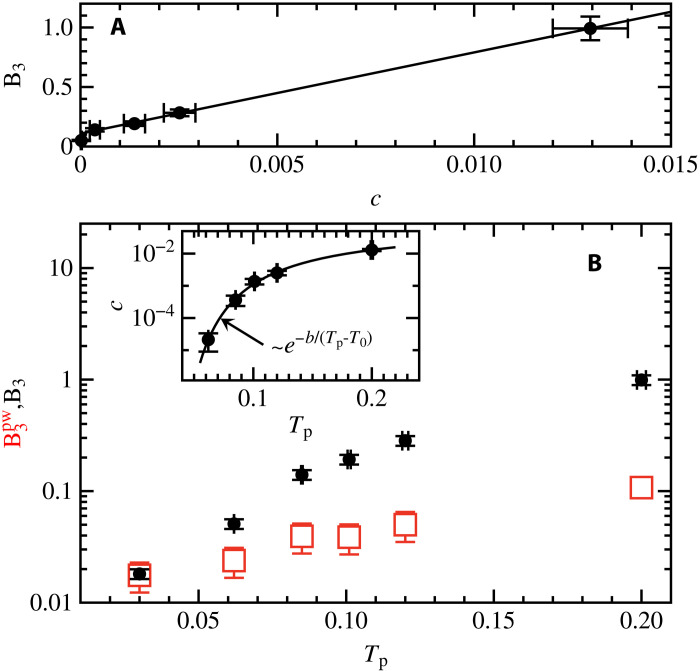
Defect contribution to damping. (**A**) Strength of sound damping, $B_{3}$, as a function of the fraction of particles within a defect. The black line is a linear fit to $T_{p}\geq 0.085$. (**B**) Parent temperature $T_{p}$ dependence of sound damping. Black circles are $B_{3}$ obtained from fits of $\Gamma_{T}=B_{3}\omega^{2}$. Red squares are $B_{3}^{\text{pw}}$ obtained from approximating the eigenvectors as plane waves and represents an approximate contribution of defect-free areas of the glass. The inset shows the $T_{p}$ dependence of the defect density, and the solid line is a fit to to $c∼e^{-b/\left(T_{p}-T_{0}\right)}$, where we find $T_{0}=0.0195\pm 0.05$.

To further motivate our definition of a defect, we plot the Rayleigh scaling coefficient $B_{3}$ found from fits of $\Gamma \left(\omega \right)=B_{3}\omega^{3}$ versus the average fraction of particles within defects, $c=\langle \sum_{n}w_{n}\rangle /N$, in Fig. 5A. We find that $B_{3}$ increases linearly with the density of particles in a defect for $T_{p}\geq 0.085$, but the y intercept is nonzero. Therefore, the defects can be a large contribution but are not the sole contribution to sound damping.

While we are only interested in defects that influence sound damping here, we note that our definition of a defect bears resemblance to defects defined by other researchers. Widmer-Cooper *et al.* (*26*) showed that particles with a large participation fraction, defined as the sum of ${|E\left(\omega_{p}\right)|}^{2}$ over the 30 lowest-frequency modes, correlated with particles that are most likely to rearrange in a supercooled fluid. Our defects would also be areas of large participation fraction. A related quantity, the vibrality $\Psi_{n}=\sum_{p}{|E_{n}\left(\omega_{p}\right)|}^{2}/\omega_{p}^{2}$, was found to be a good indicator of a structural defect responsible for plastic flow (*27*). These studies and ours suggest that defects in glasses can be identified through ${|E_{n}\left(\omega_{p}\right)|}^{2}$ for the low-frequency modes.

### Damping without defects

To estimate the contribution to damping due to areas without defects, we calculate sound attenuation in the Rayleigh regime using the plane waves of the Debye model instead of the eigenvectors of the Hessian matrix. For this calculation, we use the same non-affine force field, and we do not change the frequencies corresponding to the eigenvectors. We are interested in the effects of changing the eigenvector structure alone, which removes our defects.

Shown in Fig. 5B is the Rayleigh scaling coefficient obtained from the fits to the full theory, $B_{3}$ (black circles), and the coefficient obtained from the calculation that uses plane waves instead of the actual eigenvectors, $B_{3}^{\text{pw}}$ (red squares). For $T_{p}=0.03$, this procedure gives $B_{3}^{\text{pw}}=0.018\pm 0.002$, which is statistically the same as the one calculated using the eigenvectors of the Hessian. Therefore, defects do not play a role in sound damping for our $T_{p}=0.03$ glasses. The increase in $B_{3}^{\text{pw}}$ with increasing $T_{p}$ is due to an increase in the average magnitude of the non-affine forces with increasing $T_{p}$. With decreasing stability, the plane wave approximation becomes less accurate and largely underestimates sound damping in our poorly annealed glass.

The resulting picture is that non-affine forces, i.e., nonzero values of $\Xi_{n}$, are important in the understanding of sound damping in the harmonic approximation. The magnitude of sound attenuation is set by two contributions, one coming from defects and another one that originating from defect-free areas. The defect contribution dominates for moderately to poorly annealed glasses.

The fraction of particles in the defects depends on the glass stability, i.e., on $T_{p}$. We found that the dependence of c on $T_{p}$ can be described reasonably well by $c\propto e^{-b/\left(T_{p}-T_{0}\right)}$ over the full $T_{p}$ range (Fig. 5B, inset). We find that $T_{0}=0.0195\pm 0.005$, which is consistent with the defect density going to zero around $T_{p}=0.02$. We find that Boltzmann-like scaling $c\propto e^{-b/T_{p}}$ also provides an accurate description of the data. We note that Boltzmann-like scaling involving the parent temperature was observed for the density of quasi-localized vibrational excitations (*28*). Boltzmann-like scaling involving an effective temperature was derived for the density of shear transformation zones (*29*). While both quasi-localized excitations and shear transformation zones address similar physics as our defects, a precise relationship between them is left for future study.

To rationalize how Rayleigh scaling occurs without defects, we refer to the result of Zaccone and Scossa-Romano (*30*) who found that ${\langle 1|X|\varepsilon \left(\omega_{p}\right)\rangle }^{2}$ scales as $\omega_{p}^{2}$ within the isotropic approximation for the Hessian. We numerically verified that this is approximately accurate. Therefore, we expect that $\Gamma_{T}\left(\omega \right)\approx \left(A_{D}\bar{\epsilon }/v_{T}^{2}\right)\omega^{3}$, where $A_{D}$ is the Debye level, and $\bar{\epsilon }$ is an average $\epsilon \left(\omega_{p}\right)$ (see Materials and Methods). We find that this approximation reproduces $B_{3}$, see Table 1, but the uncertainty is larger. Thus, the existence of defects is not the necessary condition for Rayleigh scaling, and approximate sound damping can be determined from approximations to $\bar{\epsilon }$. Future theories should provide approximations to $\bar{\epsilon }$ that can be obtained from experimentally measurable quantities.

## DISCUSSION

While we are able to determine the location of damping defects, we have not determined the relationship of these defects with other theories. Defects shown in Fig. 2 resemble low-frequency quasi-localized excitations (*31*–*33*), which lead to sound damping in the soft-potential model (*9*). Using an approximation where we replace quasi-localized modes hybridized with phonons with localized modes results in sound damping scaling as the density of quasi-localized excitations $g_{s}\left(\omega \right)$. Buchenau *et al.* (*9*) derived an expression that is proportional to $g_{s}\left(\omega \right)$ for the soft potential model and thus would bear some resemblance to our theory using this approximation. Further work is needed to explore connections between the two theories. In particular, it is not clear how defect-free sound attenuation can be described within the soft potential model.

While we used properties of the eigenvectors to find defects, recent work suggests that defects may be found by examining the non-affine displacement field. It was shown in (*34*) that dislocation-like topological defects associated with plastic yielding can be identified in the non-affine displacement field for a two-dimensional glass. Recent work has expanded on the characterization of these defects and their relationship to plastic failure (*35*, *36*). Future work should examine whether these dislocation-like topological defects are also associated with areas of strong sound damping.

Kapteijns *et al.* (*15*) markedly reduced sound damping by reducing internal stresses, which, in turn, introduces a gap in the low-frequency spectrum of quasi-localized excitations (*37*). This may also remove defects. They found that sample-to-sample elasticity fluctuations could describe the relative change of sound damping. These sample-to-sample elasticity fluctuations may be related to changes in the non-affine force field, which controls defect-free sound damping.

Non-affine forces play a role both in our microscopic theory of sound attenuation and in definitions of local elastic constants (*14*). Further investigation of local elastic constants may help to establish connection between our theory and the fluctuating elasticity theory. Extending Mahajan and Ciamarra’s (*16*) comparison between sound attenuation and elastic constants fluctuations to a wider variety of systems may help clarify this issue.

Mahajan and Pica Ciamarra (*38*) studied the spatiotemporal pattern of sound damping in simulations of model three dimensional glasses. They measured a particle level attenuation and found that areas of largest attenuation were correlated with quasi-localized modes. Future work should examine the spatiotemporal character of sound damping in glasses where we do not observe defects.

## MATERIALS AND METHODS

### Simulations

We study a system of N polydisperse particles confined to a two-dimensional volume $V=L^{2}$ with $r^{-12}$ repulsive interactions that is cutoff and shifted so that the potential and its derivatives are continuous up to the second derivative. The same system was studied by Berthier *et al.* (*39*). The interaction potential is given by

$$v_{\mathrm{ij}}=v_{o}{\left(\frac{\sigma_{\mathrm{ij}}}{r}\right)}^{12}+c_{o}+c_{1}{\left(\frac{r}{\sigma_{\mathrm{ij}}}\right)}^{2}+c_{2}{\left(\frac{r}{\sigma_{\mathrm{ij}}}\right)}^{4}$$ (5)

where $\sigma_{\mathrm{ij}}=0.5\left(\sigma_{i}+\sigma_{j}\right)\left(1-0.2|\sigma_{i}-\sigma_{j}|\right)$. The potential parameter $v_{0}$ sets the units of energy, and we set Botzmann’s constant equal to one. The diameters of the particles $\sigma_{i}$ are randomly drawn from a distribution of the form $f\left(\sigma \right)=A\sigma^{-3}$ for $\sigma \in \left[\sigma_{\text{min}},\sigma_{\text{max}}\right]$ where $\sigma_{\text{min}}/\sigma_{\text{max}}=0.45$. The average diameter sets the unit of length. We cut and shift the potential at $1.25\sigma_{\mathrm{ij}}$. The results given here are for systems of N=20,000.

To create a glass, we use configurations that were equilibrated at a parent temperature $T_{p}$. We quench these configurations to a potential energy minimum using a conjugate gradient algorithm in Large-scale Atomic/Molecular Massively Parallel Simulator (LAMMPS) (*40*). The stability of the glass is determined by its parent temperature. We examine in detail three parent temperatures, a poorly annealed glass at $T_{p}=0.2$, an intermediate parent temperature $T_{p}=0.101$, and a very stable glass $T_{p}=0.03$.

### Damping theory

We consider the harmonic approximation where the equation of motion is

$$\partial_{t}^{2}u_{n}={\ddot{u}}_{n}=-\sum_{m}H_{\mathrm{nm}}\cdot u_{m}$$ (6)

where $H_{\mathrm{nm}}$ is the Hessian calculated at the inherent structure positions $\left\{R_{n}\right\}$, and $u_{n}$ is the displacement of particle n from $R_{n}$. The initial conditions in the simulations are ${\dot{u}}_{n}\left(t=0\right)=a\text{sin}\left(k\cdot r_{n}\right)$, $u_{n}=0$, and a⋅k=0 for a transverse wave and a parallel to k for a longitudinal wave. We determine sound damping in simulations by fitting the envelope of $C_{k}\left(t\right)=\left[\dot{u}\left(t\right)\cdot \dot{u}\left(0\right)\right]/\left[\dot{u}\left(0\right)\cdot \dot{u}\left(0\right)\right]$ to $e^{-\Gamma t/2}$ as was done in previous work (*11*).

The theory (*24*) is formulated such that the initial conditions are $u_{n}\left(t=0\right)=be^{-ik\cdot R_{n}}$ and ${\dot{u}}_{n}\left(t=0\right)=0$. We define a two-dimensional vector $e_{n}$ such that $e_{n}\cdot e_{n}=1$ and is identical for each n. Solving the equations of motion (Eq. 6) is equivalent to solving $\partial_{t}^{2}|1\left(t\right)\rangle =-H\left(k\right)|1\left(t\right)\rangle$, where $H_{\mathrm{nm}}\left(k\right)=H_{\mathrm{nm}}e^{ik\cdot \left(R_{n}-R_{m}\right)}$, with the initial condition $|1\left(t=0\right)>=|1\rangle =N^{-1/2}\left(e_{1},\ldots ,e_{N}\right)$.

In practice, the low-frequency limit of transverse sound damping $\Gamma_{T}\left(\omega \right)$ is calculated using the distribution (*24*)

$$\Gamma_{T}\left(\omega \right)=\frac{\omega^{2}}{v_{T}^{2}}\frac{1}{\delta \omega }\sum_{\omega_{p}\in \left\{\omega -\delta \omega /2,\omega +\delta \omega /2\right\}}\epsilon \left(\omega_{p}\right)$$ (7)

where $\epsilon \left(\omega_{p}\right)=\left(\pi \right)/\left(2\omega_{p}^{2}\right){|\langle 1|X|ℰ\left(\omega_{p}\right)\rangle |}^{2}$ and $c_{T}$ is the transverse speed of sound. In practice, one must include frequencies where plane wave–like modes exist in the finite sized system. In Eq. 7, $|\varepsilon \left(\omega_{p}\right)\rangle$ is a normalized eigenvector of ℋ with eigenvalue $\omega_{p}^{2}$. When we refer to a mode or vibrational mode in this work, we are always referring to an eigenvector of the Hessian.

To determine X, we first set $e_{n}=\left(1,0\right)$, define the matrix $X_{\mathrm{nm}}^{1}=H_{\mathrm{nm}}\left(Y_{n}-Y_{m}\right)$, and determine the distribution given in Eq. 7. We then set $e_{n}=\left(0,1\right)$, define the matrix $X_{\mathrm{nm}}^{2}=H_{\mathrm{nm}}\left(X_{n}-X_{m}\right)$, and determine the distribution given by Eq. 7. We then average the distributions and fit the resulting distribution to Aω over a frequency range where Rayleigh scaling is observed. The damping coefficient is given by $A\omega^{3}/v_{T}^{2}$. The results of these fits are given in Table 1. We examine distributions with δω ranging from 0.05 to 0.15. Since we are fitting a range of frequencies, we find that the bin size makes little difference. We find the speed of sound by using the theory given by Szamel and Flenner (*24*).

There are several important aspects to Eq. 7. First, it is a weighted distribution that would be proportional to the density of states if the weights $\epsilon \left(\omega_{p}\right)$ were all equal. Therefore, the density of states influences the frequency dependence of sound damping. Second, the weights $\epsilon \left(\omega_{p}\right)$ can be thought of as different contributions from the vibrational mode $|\varepsilon \left(\omega_{p}\right)\rangle$, and only modes around the frequency of the sound wave contribute to sound damping.

While $\epsilon \left(\omega_{p}\right)$ differs by orders of magnitude depending on the details of the eigenvector and X, on average, it does not grow or decrease over the Rayleigh scaling regime. Hence, we can approximate the value of the distribution by calculating $\bar{\epsilon }=\left(2N/{\mathbb{N}}_{p}\right)\sum_{p}^{{\mathbb{N}}_{p}}\epsilon \left(\omega_{p}\right)$ where the sum is taken over the range of frequencies where Rayleigh scaling is observed. For *T*_p_ = 0.2, we took an average up to ω = 0.24; for *T*_p_ = 0.101, we averaged up to ω = 0.55; and for *T*_p_ = 0.03, we averaged up to ω = 1.4. As long as we included the first 24 modes per configuration in the average, the average varies by less than 20%, which is close to the uncertainty in all our calculations.
